# Concurrent infections of cells by two pathogens can enable a reactivation of the first pathogen and the second pathogen's accelerated T-cell exhaustion

**DOI:** 10.1016/j.heliyon.2022.e11371

**Published:** 2022-11-30

**Authors:** Kevin Roe

**Affiliations:** San Jose, California, USA

**Keywords:** T-cell exhaustion, Accelerated T-cell exhaustion, T-cell suppression, Viral infections, Bacterial infections, Protozoan infections

## Abstract

When multiple intracellular pathogens, such as viruses, bacteria, fungi and protozoan parasites, infect the same host cell, they can help each other. A pathogen can substantially help another pathogen by disabling cellular immune defenses, using non-coding ribonucleic acids and/or pathogen proteins that target interferon-stimulated genes and other genes that express immune defense proteins. This can enable reactivation of a latent first pathogen and accelerate T-cell exhaustion and/or T-cell suppression regarding a second pathogen. In a worst-case scenario, accelerated T-cell exhaustion and/or T-cell suppression regarding the second pathogen can impair T-cell functionality and allow a first-time, immunologically novel second pathogen infection to escape all adaptive immune system defenses, including antibodies. The interactions of herpesviruses with concurrent intracellular pathogens in epithelial cells and B-cells, the interactions of the human immunodeficiency virus with *Mycobacterium tuberculosis* in macrophages and the interactions of *Toxoplasma gondii* with other pathogens in almost any type of animal cell are considered. The reactivation of latent pathogens and the acceleration of T-cell exhaustion for the second pathogen can explain several puzzling aspects of viral epidemics, such as COVID-19 and their unusual comorbidity mortality rates and post-infection symptoms.

## 1 Introduction

When multiple intracellular pathogens, such as viruses, bacteria, fungi and protozoan parasites, infect the same host cell, they can help each other by impairing cellular defenses. One pathogen can help a second pathogen or they can both help each other.

One aim of this paper is to consider the possible interactions between concurrent intracellular pathogens in a broader context, especially when one pathogen worsens the consequences of another pathogen infection. This leads to the question of how pathogens can help each other against immune defenses.

## 2 Discussion

### 2.1 Intracellular pathogens subvert innate and adaptive immune defenses

Intracellular pathogens can subvert intracellular and adaptive immune defenses using proteins and by using or synthesizing short non-coding ribonucleic acids (RNAs) [1, 2]. Several viral pathogens, including Epstein-Barr virus (EBV), Kaposi sarcoma-associated herpesvirus (KSHV) and human cytomegalovirus (HCMV), are known to use or synthesize multiple types of short non-coding RNAs to evade or disable several intracellular defenses [1, 2]. The extensively studied herpesvirus EBV is known to use or synthesize several dozen types of microRNAs (usually consisting of 19–22 nucleotides) that can regulate hundreds of host cell genes by interfering with gene expression and messenger RNA processing, thus allowing EBV to evade or disable several immune defenses [1]. Some non-coding RNAs can bind to infected host cell signal transducer and activator of transcription (STAT) transcription factors and messenger RNAs to regulate gene expression of interferon-stimulated genes (ISG) and thereby enable these pathogens to evade recognition by innate immune cells including natural killer cells (NK-cells) and adaptive immune cells including thymic cells (T-cells) [1, 2]. Not only would ISG disruption help EBV, KSHV and HCMV evade recognition by innate immune cells (NK-cells) and adaptive immune cells (T-cells) [1, 2], it would also interfere with ISG cellular defenses against any other intracellular pathogen concurrently infecting the same host cell. This would apply to bone marrow cells (B-cells), endothelial cells and epithelial cells infected by herpesviruses, and the pathogens that could benefit from herpesvirus co-infections include several viruses, bacteria, fungi or protozoan parasites.

The pro-pathogenic effects of non-coding RNAs can occur in all cycles of a cellular infection. For example, EBV viral non-coding RNAs can be synthesized and released in the prelatent time after infection, during viral latent infections and during the lytic stage for release of viral replicants [1]. In addition, the inhibition of cellular immune defenses against multiple pathogens, including pathogenic use of non-coding RNAs and/or proteins against interferon-stimulated genes, can also facilitate the reactivation of latent intracellular pathogens [1, 2]. One very significant interaction of intracellular pathogens is the interaction of human immunodeficiency virus (HIV) and *Mycobacterium tuberculosis*, which is estimated to infect one fourth of the global human population, wherein *M. tuberculosis* reactivation by HIV is estimated to cause one third of global HIV mortalities [3].

### 2.2 *Mycobacterium tuberculosis* - an intracellular pathogen that targets macrophages

*M. tuberculosis* is an intracellular pathogen which can infect macrophages and other cell types, usually residing in a membrane-bound vacuole, the phagosome, and/or it can also escape into the cytosol at late stages of infection [4]. *M. tuberculosis* avoids death by autophagolysosomes (autolysosomes) inside macrophages by interfering with macrophage phagosome maturation and the macrophage endocytic machinery to block phagosome fusion with late endosomes and lysosomes [4, 5, 6]. *M. tuberculosis* can also resist acid stress and reactive oxygen and nitrogen oxidative species released by cells during infections [4, 6].

### 2.3 *Mycobacterium tuberculosis* bacteria and human immunodeficiency viruses

One conservative estimate is that worldwide, two billion people are latently infected with *M. tuberculosis*, ten million people are diagnosed with active or reactivated *M. tuberculosis* (TB) each year and it's yearly mortality world-wide is 1.5 million deaths [7]. HIV-induced impairment of innate immune system macrophages and monocytes also promotes active and reactivated TB infections in *M. tuberculosis* infected individuals [7]. Individuals concurrently infected with HIV and TB have a greatly elevated risk (estimated to be at least 16X and perhaps as high as 27X) of reactivating TB relative to individuals without HIV [7]. One fundamental reason is that HIV infection causes CD4+ T-cell depletion [8], whereas these T-cells are essential to controlling TB infections [7].

Furthermore, HIV infection also significantly reduces anti-pathogenic monocyte chemotaxis (attraction of immune cell monocytes) and monocyte oxidative burst capabilities [7]. HIV infection also reduces the expression of Toll-like receptors (TLRs) essential for recognizing pathogens including *M. tuberculosis* [7, 9]. Experimental HIV infections of human monocyte-derived macrophages (hMDMs) promoted an inflammatory M1 macrophage phenotype and altered macrophage metabolism [7, 10]. And hMDMs experimentally infected with HIV also prevent activation of transcription factor STAT5A, a transcription factor essential in controlling TB [7]. Additionally, HIV infected macrophages also have inhibited production of essential antibacterial cytokines, such as granulocyte-macrophage colony-stimulating factor GM-CSF [11]. Furthermore, HIV can also infect alveolar (lung) macrophages [12] and utilize these macrophages as HIV reservoirs [10, 13]. As a consequence, HIV-infected alveolar macrophages demonstrate inhibited phagocytosis of cells [7].

Therefore, host immune cells including monocytes and macrophages co-infected with HIV and TB exhibit numerous impaired immune defenses for the benefit of both pathogens. Furthermore, other types of host cells in addition to macrophages can be infected by intracellular pathogens, not only viruses, fungi and bacteria, but also thousands of species of protozoan parasites of the phylum Apicomplexa, which is best exemplified by the broadly infective and highly versatile pathogen *Toxoplasma gondii* [14, 15, 16]. A summary of *T. gondii* capabilities is worthwhile.

### 2.4 The very pervasive intracellular pathogen *Toxoplasma gondii*

*T. gondii* is a pervasive, skillful and manipulative protozoan parasite, and a common co-infecting intracellular pathogen in animal and mammal cells, since it is a very prevalent intracellular pathogen world-wide [14, 15, 16]. *T. gondii* is also estimated to infect approximately one third of the global human population [14, 15, 16, 17]. *T. gondii* has several genetic strains (haplogroups) with enormous differences in immunological effects, and Type I is the most dangerous genetic strain for humans, since it can be more than five orders of magnitude more virulent than the milder *T. gondii* genetic strains [14, 17]. *T. gondii* can be transmitted by ingestion of food or water, by tissue or organ transplants and by congenital transmission from mother to fetus [14, 17]. *T. gondii* can maintain life-long latent intracellular infections by bradyzoite cysts, especially inside muscle cells and brain cells [14, 15, 16, 17]. Presently there are treatments for active infections, but there are no treatments for the cysts and there is no vaccine for either active or latent *T. gondii* infections [14, 15, 16, 17]. During its active phase, or during its reactivated phase after latency, it can cause severe encephalitis, hepatitis, or myocarditis in individuals immuno-compromised by other pathogens, such as HIV and probably SARS-CoV-2 [14, 18]. *T. gondii* reactivations would be a plausible cause for several puzzling cases of hepatitis seen in children or adults after SARS-CoV-2 infections where hepatitis viruses were absent. This will be discussed later.

### 2.5 Concurrent pathogen interactions - potentially accelerated T-cell exhaustion

Another way that a first pathogen can assist a second later pathogen with potentially disastrous consequences is by facilitating or even accelerating T-cell exhaustion and/or T-cell suppression for the second pathogen. T-cell exhaustion will be briefly reviewed prior to a discussion of it's acceleration.

### 2.6 A brief review of T-cell exhaustion and T-cell suppression

T-cell exhaustion or suppression are basically indicating a major inhibition of T-cell functions for CD4^+^ and/or CD8^+^ T-cells [17, 19, 20]. T-cell exhaustion is usually caused by cancers or chronic pathogen infections [19, 20, 21], and results from continuous long duration antigen exposures [17, 20, 21, 22, 23].

In one of the early studies of T-cell exhaustion in mice, if the antigen exposure from lymphocytic choriomeningitis virus (LCMV) was chronic and continuous for over two to four weeks, T-cell exhaustion characteristics first appeared at about two weeks after infection and an irreversible full T-cell exhaustion phenotype was seen approximately four weeks after infection [24]. It is also known that T-cell exhaustion can occur even from continuous low levels of antigens, and T-cell exhaustion usually comprises T-cell metabolic exhaustion and mitochondrial dysfunctions, with reduced proliferation and effector functions and possibly diminished T-cell numbers [23, 24]. T-cell exhaustion can be induced by chronic and/or latent infections of fungal, bacterial, protozoan pathogens or viral pathogens, such as hepatitis B virus or hepatitis C virus [25–27]. Inflammation can also occur, and it is facilitated by the release of numerous pro-inflammatory cytokines and chemokines [28].

### 2.7 T-cell inhibitory receptors

T-cell exhaustion impairs CD4^+^ T-cell and CD8^+^ T-cell reactions to pathogen infections [20, 25, 27]. Numerous T-cell receptors are involved in T-cell exhaustion, including co-stimulatory and inhibitory receptors [25]. Higher numbers of multiple inhibitory receptors correlate to an increased level of T-cell exhaustion [25]. The T-cell inhibitory receptors include the programmed cell death protein 1 (PD-1), lymphocyte activation gene 3 protein (LAG-3), CD244, CD160, T-cell immunoglobulin domain and mucin domain-containing protein 3 (TIM-3), and cytotoxic T-lymphocyte-associated protein 4 (CTLA4) [25, 26, 27, 28, 29, 30]. The inhibitory receptors have a higher level of expression corresponding to a higher level of T-cell exhaustion [30]. For instance, chronic viral infections can result in extensive expressions of the inhibitory TIM-3 receptor on severely exhausted T-cells [30].

However, several inhibitory receptors are also present during the differentiation or activation of T-cells, and their presence during active infections does not always result from T-cell exhaustion [31]. Furthermore, during acute infections, the cytokine environment can significantly increase T-cell inhibitory receptor expression in 24 h, and induce substantially higher numbers of inhibitory receptors in 48 to 72 h [31].

T-cell exhaustion is reversible and/or can be avoided by blockades of specific co-stimulatory and inhibitory T-cell receptors, including a blockade of the interleukin-10 (IL-10) receptor, a blockade of the PD-1 receptor on CD8^+^ T-cells, and a blockade of CD4^+^ T-cell signaling by means of type I interferons (interferon α and β) [20, 27, 29]. Type I interferons α and β are essential cytokines in the early stages of viral infection for the activation and differentiation of CD8^+^ T-cells, but a long duration release can induce CD4^+^ T-cell exhaustion [20, 21, 29]. CD4^+^ T-cell exhaustion results in more CD8^+^ T-cell exhaustion, because of a reduction in interleukin-21 that is secreted by CD4^+^ T-cells [20, 21, 29]. And decreased functionality resulting from CD4^+^ T-cell exhaustion will reduce interferon-γ levels, essential for mobilizing intracellular defenses against acute and chronic pathogen infections [17, 32].

### 2.8 A first pathogen's T-cell exhaustion can facilitate a second pathogen's T-cell exhaustion

A fundamental question concerns how T-cell exhaustion in antigen-specific T-cells could cause T-cell exhaustion in other T-cells specific to other pathogens. There are pathways enabling concurrent pathogens to induce T-cell exhaustion and/or T-cell suppression [20, 33, 34, 35, 36]. As previously discussed, ligand binding and activation of inhibitory receptors and/or desensitization of T-cells' co-stimulatory receptors can cause T-cell exhaustion [20, 21, 24]. As an example, chronic infections of *T. gondii* numerically increase the number of inhibitory PD-1 receptors on T-cells and cause higher numbers of programmed cell death ligand 1 (PD-L1) ligands on *T. gondii* infected cells [37]. These higher numbers of inhibitory receptors and ligands facilitate their binding and the activation of the inhibitory PD-1 receptors on T-cells by cells infected by both *T. gondii* and a second pathogen, and the activation of the inhibitory receptors on their corresponding subsets of T-cells could result in CD8^+^ T-cell exhaustion for both the first and second pathogens.

### 2.9 T-cell exhaustion and/or T-cell suppression have numerous pathways

T-cell exhaustion and/or T-cell suppression can result from cytokines and cells that impair T-cell functionality, such as the cytokines IL-10 and transforming growth factor-β (TGF-β), and the enzyme IDO [30]. T-cell exhaustion and/or T-cell suppression can result from type I interferons α and β cytokines [30], from dendritic cells, macrophages and B cells capable of acting as antigen presenting cells secreting the cytokines IL-10 and TGF-β and the enzyme IDO, and from myeloid-derived suppressor cells [30, 33, 34, 35, 36]. Therefore, there are numerous pathways for T-cell exhaustion and/or T-cell suppression to be shared by a first pathogen and a second pathogen.

Regulatory T-cells (T_REG_ cells) also participate in T-cell exhaustion and/or T-cell suppression, such as by maintaining CD8^+^ T-cell exhaustion [24, 30]. T_REG_ cells impair antigen-specific T-cells by the secretion of inhibitory cytokines including IL-10, interleukin-35 (IL-35), and TGF-β, [30, 38]. T_REG_ cells also use their IL-2 receptors (CD25) to lower background levels of IL-2 and decrease T-cell replication [30, 38].

### 2.10 T-cell exhaustion acceleration can cause serious second pathogen infections

T-cell exhaustion typically requires several weeks to become significant [24], and the extent of T-cell exhaustion can worsen during long duration infections and chronic antigen titers [17, 29]. It is also possible for a long duration chronic first pathogen infection to accelerate T-cell exhaustion for a second pathogen by causing T-cells to express multiple inhibitory receptors (e.g., PD-1) and by causing infected host cells to express inhibitory ligands (e.g., PD-L1) for those inhibitory receptors [37]. Later, the second pathogen infection, possibly a pathogen that produces large antigen titers, can utilize the cytokine environment, and utilize the expression of inhibitory ligands by the same infected cell, to accelerate a T-cell exhaustion towards its own antigens. This can inhibit T-cell actions towards the second pathogen, and could enable the second pathogen to overcome the adaptive immune responses of the host [17, 39]. This outcome would be especially dangerous for immunologically novel secondary infections in which antibodies (produced from B-cells by CD4^+^ T-cell assistance) are inadequate in numbers and/or inadequate in affinity selection/maturation from somatic hypermutation to control the second pathogen [39]. An observed result may be high mortality rate viral epidemics, in which the second pathogen infection produces high antigen titers, accelerated T-cell exhaustion occurs, and the second pathogen quickly overcomes the adaptive immune system of the host.

The timing of conventional T-cell exhaustion was analyzed most extensively in murine experiments involving lymphocytic choriomeningitis virus (LCMV) [22, 23, 24, 25]. If it is plausibly assumed that other viruses in mice or humans have approximately the same timing regarding conventional T-cell exhaustion, and infection mortality is approximately contemporaneous with fully developed T-cell exhaustion, the extensively studied COVID-19 pandemic mortality can provide quantitative timing data to help distinguish between the fatal consequences of conventional T-cell exhaustion or accelerated T-cell exhaustion [40, 41, 42, 43].

Specifically, T-cell exhaustion has been observed in severe cases of COVID-19 [40, 41]. T-cell exhaustion can be the fundamental driver of COVID-19 patient mortality, such as exhaustion of CD8^+^ T-cells and CD4^+^ T-cells that would have controlled the virus [40, 41, 42, 43]. Exhaustion can also affect follicular helper CD4^+^ T-cells, mainly in germinal centers in lymph nodes and the spleen, which are critical to antibody affinity maturation, isotype switching, generation of memory B-cells and B-cell differentiation into immunoglobulin (antibody) secreting plasma cells [44]. Accelerated T-cell exhaustion could be particularly dangerous in first-time infections of the second pathogen, if germinal center follicular helper CD4^+^ T-cells are inhibited [45]. This can cause antibodies from B-cells to lack optimal antibody isotype switching and prevent antibody improvements by somatic hypermutation and affinity selection/maturation to better target the second pathogen [39, 45]. It is documented that patients with severe COVID-19 have higher proportions of less-effective IgM immunoglobulins, in comparison to immunoglobulins seen in control patients or mild COVID-19 patients [44]. This suggests that follicular helper CD4^+^ T-cell exhaustion is a major factor in causing severe COVID-19 cases and mortality [43, 44, 45].

### 2.11 Are some cases of COVID-19 mortality caused by accelerated T-cell exhaustion?

If conventional T-cell exhaustion or accelerated T-cell exhaustion are causing severe COVID-19 outcomes including mortality, the number of days required for conventional T-cell exhaustion, accelerated T-cell exhaustion and patient mortality should be considered and compared. Statistical analysis of 8873 COVID-19 patient mortality cases in the database of Johns Hopkins University indicated a relatively brief median time from COVID-19 symptom diagnosis to death (16.33 days for male patients and 17.67 days for female patients), over a time period covering cases of the Alpha, Beta, Delta and Omicron variants of SARS-CoV-2 [46].

In addition, a statistical analysis of January to December 2020 case data (during the Alpha and Beta variants of SARS-CoV-2) of the time span starting from COVID-19 diagnosis to death for 816 case fatalities determined that the COVID-19 diagnosis to death mean time span was approximately 18 days [47]. The time required for COVID-19 symptoms to produce a COVID-19 diagnosis is comparatively short, and it will be considered negligible in order to simplify comparing the case mortality studies. Two other conclusions from statistical analysis of the 816 COVID-19 fatalities were that the probability of mortality increased in older age groups, and yet the time span from COVID-19 diagnosis to death was virtually unrelated to a patient's age, which is surprising and remarkable [47]. This relatively age-independent time span implies that the means of death is primarily due to the SARS-CoV-2 virus effects on the immune system cells, e.g., T-cell exhaustion, and not exclusively due to the age-related immune system degradation in the deceased patients.

These time spans from diagnosis to death (∼16 to ∼18 days) are relatively short, because these median time spans from COVID-19 diagnosis to death are significantly less than what would be expected from deaths fundamentally resulting from conventional T-cell exhaustion. But this conclusion depends on whether there is a significant difference in the time required to see T-cell exhaustion induced by the viral pathogens SARS-CoV-2 or LCMV.

The time delay to see T-cell exhaustion from SARS-CoV-2 or other viral pathogens should be approximately the same, since this time delay fundamentally depends on the T-cells and host cells, including the time delay for inhibitory receptor expression on T-cells and the time delay for infected host cell expressions of inhibitory ligands for the T-cell inhibitory receptors. In mice, after LCMV infection, the earliest T-cell exhaustion characteristics occurred ∼15 days post-infection and irreversible complete T-cell exhaustion occurred in ∼30 days [48].

### 2.12 Significant COVID-19 case mortality is plausibly caused by accelerated T-cell exhaustion

The time from infection with SARS-CoV-2 to death is difficult to measure, whereas measuring the time from symptoms and diagnosis of COVID-19 to death is simpler, if an adjustment factor can be determined to compensate for the time delay from infection to symptoms and diagnosis. A recent study analyzed the incubation period for COVID-19, defined as the time from exposure (∼infection) to the time of appearance of COVID-19 symptoms, for the Alpha, Beta, Delta and Omicron variants of SARS-CoV-2 [49]. This study determined that the mean incubation period of COVID-19 was 5 days (95% confidence interval (CI) 4.94–5.06 days) for the Alpha variant, 4.5 days (95% CI, 1.83–7.17 days) for the Beta variant, 4.41 days (95% CI, 3.76–5.05 days) for the Delta variant, and 3.42 days (95% CI, 2.88–3.96 days) for the Omicron variant [49]. Five days could be subtracted for a conservative delay time after exposure to SARS-CoV-2 to result in COVID-19 symptoms and diagnosis. The subtraction of this adjustment factor suggests that complete conventional T-cell exhaustion would appear at about 25 days after COVID-19 symptoms and diagnosis [50]. Accelerated T-cell exhaustion is one plausible explanation for the difference between the expected 25 days and the observed median times of ∼16 to ∼18 days and observed average of 18 days for COVID-19 fatalities primarily due to the variants of SARS-CoV-2 [46, 47, 50]. Some skew in the timing statistics could be due to quicker fatalities resulting from comorbidities, but in approximately 50% of the fatal COVID-19 cases analyzed, accelerated T-cell exhaustion can better explain the timing of the observed fatalities compared to conventional T-cell exhaustion.

### 2.13 T-cell exhaustion and Long COVID

A long duration of lymphocyte exhaustion for T-cells and NK-cells was also seen in COVID-19 survivors, even several weeks post-infection [42]. Such multiple lymphocyte exhaustions could be a major causational factor for the COVID-19 post-infection symptoms collectively called Long COVID [42, 51].

In one case, a first pathogen infects a first subset of host cells and achieves T-cell exhaustion regarding the first pathogen, and a second pathogen infects a second subset of host cells and achieves T-cell exhaustion regarding the second pathogen. This situation would not facilitate reuse of the inhibitory ligands by the second pathogen, but the pre-existing inflammation and cytokine environment created in the T-cell exhaustion for the first pathogen could facilitate the second pathogen in more quickly achieving T-cell exhaustion. The delay time for the second pathogen to achieve T-cell exhaustion could be determined by these requirements: (1) the time needed for inhibitory ligands to be expressed by the host cell infected by the second pathogen, if not already expressed, and (2) the time (24 h–72 h) required for the T-cells for the second pathogen to express several inhibitory receptors [31].

Table 1 summarizes the steps by which a first pathogen can create long duration antigen levels and inflammation to achieve T-cell exhaustion for itself, and assist a virulent second pathogen to achieve an accelerated T-cell exhaustion.

**Table 1:** The steps for a pathogen to induce chronic infections, T-Cell exhaustion and accelerated T-Cell exhaustion for a second pathogen.

1. A first pathogen infection begins in host cells.
2. The first pathogen manipulates the immune defenses of the host cell.
3. The first pathogen establishes a chronic infection after overcoming the immune defenses.
4. A long duration first pathogen chronic infection induces T-cell exhaustion regarding the first pathogen, including expression of inhibitory ligands on the infected host cells and expression of inhibitory receptors on the T-cells for the first pathogen.
5. Some time later the same host cells are infected by a second pathogen, possibly novel and virulent.
6. The second pathogen infection is facilitated by the disablement of some or all of the immune defenses of the host cells.
7. Existing inhibitory ligands on the same host cells can be used by the second pathogen to induce an accelerated T-cell exhaustion towards the second pathogen.
8. If the remaining adaptive immune system defenses are unprepared for the novel, first-time infection by the second pathogen, then the second pathogen can potentially outpace the immune response of the host.
9. If no external treatment stops the second pathogen, this second infection can have a severe outcome. If the patient survives, immune dysfunctions induced from the second pathogen can reactivate the first pathogen to cause severe post-infection symptoms. This can explain several puzzling symptoms seen later in survivors after epidemics.

One example would be a first pathogen, such as a protozoan parasite or bacteria infecting host cells, and the second pathogen is a virulent virus that can infect the same host cells already infected by the first pathogen. The already extensive expression of inhibitory ligands on the same host cells facilitates the reuse of the inhibitory ligands by the second pathogen, and the pre-existing cytokine environment created in the T-cell exhaustion for the first pathogen could also more quickly create T-cell exhaustion for the second pathogen. The delay time to achieve T-cell exhaustion for the second pathogen may not require the time required for inhibitory ligands to be expressed by the host cell infected by the second pathogen. The delay time for T-cell exhaustion may be the relatively shorter time (24 h–72 h) required for the T-cells for the second pathogen to express several inhibitory receptors [31]. This short time may be fatal, because an accelerated T-cell exhaustion for the second pathogen could precede an effective antibody defense against the second pathogen by means of affinity selection and antibody isotype switching [39].

**Figure 1:**
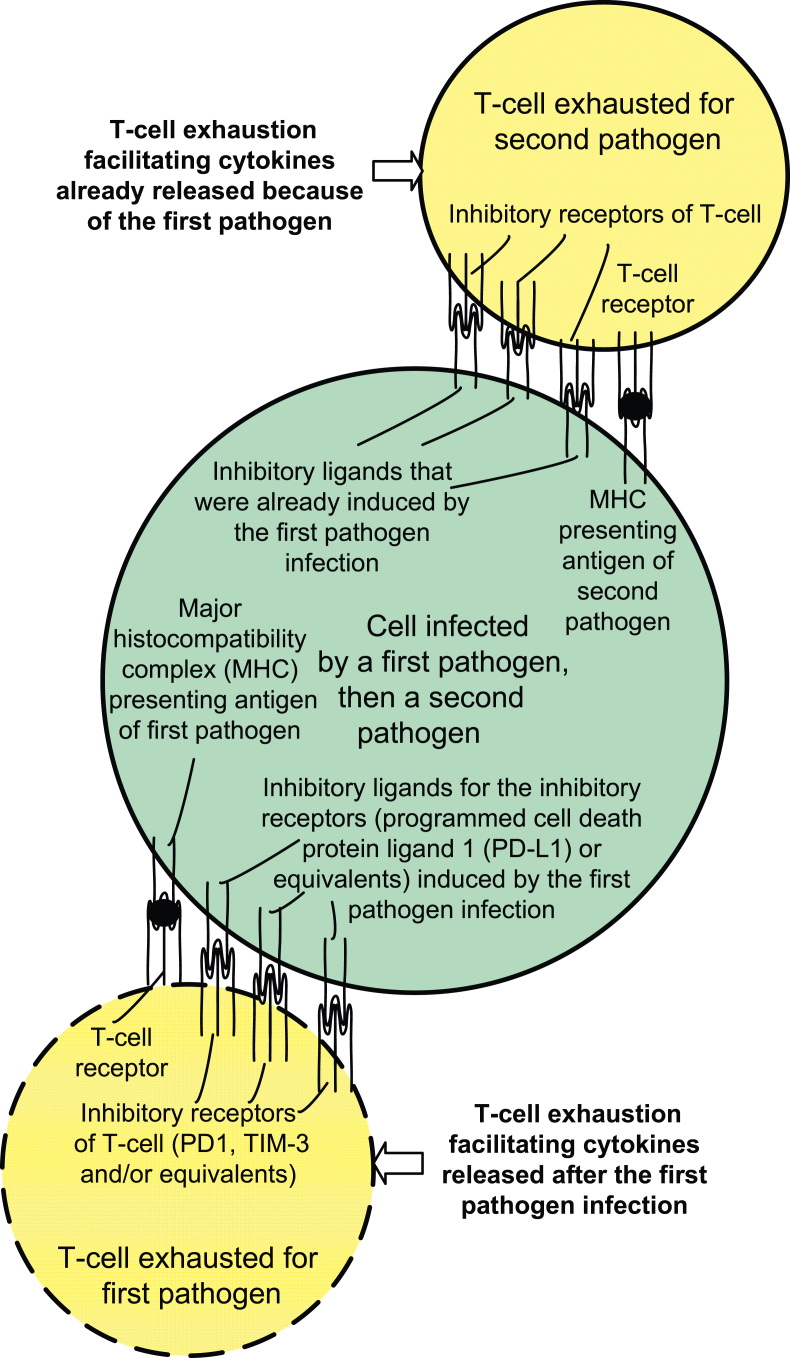
Is a simplified conceptual diagram of a cell infected by a first latent intracellular pathogen and a second pathogen which enables the reuse of the inhibitory ligands by the second pathogen to potentially assist the second pathogen in achieving an accelerated T-cell exhaustion.

Figure 1 is a simplified diagram of a cell infected by a first latent intracellular pathogen and a second pathogen, such as a virus. Concurrent intracellular infections enable the reuse of the inhibitory ligands by the second pathogen, and the pre-existing inflammation and cytokine environment created by the first pathogen could enable the second pathogen to achieve an accelerated T-cell exhaustion before the time normally required after infection. The two illustrated T-cells subject to exhaustion are also shown, but it should be noted that they would not have to be present at the same time.

If the host is able to survive the accelerated T-cell exhaustion for the second pathogen and a faster-paced second pathogen infection from the weakened T-cell functionality, the first pathogen is a threat. If the first pathogen was a latent infection, T-cell exhaustion for the first pathogen can enable a reactivation of the latent first pathogen infection [17]. Such a reactivation could potentially have severe consequences (such as encephalitis, hepatitis, or myocarditis, in the case of toxoplasmosis by *T. gondii*) [18]. Mother-to-fetus transmission of *T. gondii* and other protozoan parasites, or other maternally transmitted pathogens, or oral infections from food and water [17], later reactivated by pediatric viral infections, could plausibly explain the recent world-wide pediatric cases of hepatitis seen in children lacking the viruses that normally cause hepatitis, if the latent pathogen reactivations were facilitated by recent infections of COVID-19 [52, 53, 54].

## 3 Conclusion

Interactions between two intracellular pathogens can have potentially severe consequences, including reactivation of a first pathogen or an accelerated T-cell exhaustion for a second pathogen. One potential danger from T-cell exhaustion is that a long duration chronic and/or latent first pathogen infection can create a cytokine environment where T-cells express multiple inhibitory receptors and infected cells express multiple inhibitory ligands for those inhibitory receptors. Then a novel second pathogen infection, particularly a virulent pathogen that creates large antigen titers, can achieve an accelerated T-cell exhaustion to potentially escape T-cell control. This acceleration of T-cell exhaustion is facilitated when the first and second pathogens infect the same cells, allowing reuse of the already expressed inhibitory ligands of the infected cells. One already observed result of accelerated T-cell exhaustion may be the comparatively high mortality rates seen in some viral epidemics, possibly including COVID-19, where the second pathogen infection causes continuous long duration high antigen titers and an accelerated T-cell exhaustion and overwhelms a host's remaining adaptive immune system defenses. Reactivation of a latent first pathogen in survivors is also possible and could explain many post-infection symptoms seen in viral epidemics such as COVID-19. For instance, congenital or conventional *T. gondii* infections could be reactivated to cause several puzzling cases of encephalitis, hepatitis, or myocarditis exhibited by children or adults after SARS-CoV-2 infections.

## Ethics approval

No ethical approval was required as this is a review article with no original research data.

## Declarations

### Author contribution statement

All authors listed have significantly contributed to the development and the writing of this article.

### Funding statement

This research did not receive any specific grant from funding agencies in the public, commercial, or not-for-profit sectors.

### Data availability statement

No data was used for the research described in the article.

### Declaration of interest’s statement

The authors declare no conflict of interest.

### Additional information

No additional information is available for this paper.
